# Increased expression of CX43 on stromal cells promotes leukemia apoptosis

**DOI:** 10.18632/oncotarget.6249

**Published:** 2015-10-27

**Authors:** Shijie Yang, Qin Wen, Yao Liu, Cheng Zhang, Maihong Wang, Guo Chen, Yi Gong, Jiangjian Zhong, Xuelian Chen, Andres Stucky, Jiang F. Zhong, Xi Zhang

**Affiliations:** ^1^ Ostrow School of Dentistry and Department of Pediatrics, School of Medicine, University of Southern California, Los Angeles, CA, USA; ^2^ Department of Hematology and Blood Transfusion, Xinqiao Hospital, Third Military Medical University, Chongqing, P. R. China; ^3^ Z-Genetic Medicine LLC, Temple City, CA, USA

**Keywords:** leukemia, bone marrow stromal cells, apoptosis

## Abstract

Connexin 43 (Cx43) induced apoptosis has been reported in solid tumors, but the effect of Cx43 expressed by bone marrow stromal cells (BMSC) in leukemia has not been fully investigated. Manipulating Cx43 expression could be a potential therapeutic strategy for leukemia. Here, we investigate the effect of Cx43 expressed by BMSCs (human Umbilical Cord Stem Cells over-expressed CX43, Cx43-hUCSC) on leukemia cells. When co-cultured with Cx43-hUCSC, leukemia cells show significant lower growth rate with increasing apoptosis activity, and more leukemia cells enter S phase. Functional assays of fluorescence recovery after photo bleaching (FRAP) showed improved gap junctional intercellular communication (GJIC) on leukemia cells when co-cultured with Cx43-hUCSC (*p* < 0.01). In a mouse minimal disease model, the mean survival time and mortality rate were significantly improved in mice transplanted with Cx43-hUCSC. Our results indicate that Cx43 expressed by BMSC induces apoptosis on leukemia cells. Small molecules or other pharmaceutical approaches for modulating Cx43 expression in BMSCs could be used for delaying relapse of leukemia.

## INTRODUCTION

Although the survival rate of leukemia has been greatly improved due to new drugs, relapse of leukemia from minimal residue disease (MRD) remains a clinical challenge. The remaining leukemia stem cells in bone marrow (BM) following radiotherapy and/or chemotherapy are the primary cause for minimal residual disease (MRD) [1, 2]. Therefore, modifying the BM microenvironment upon which leukemic stem cells depend for existence is a potential novel therapeutic strategy with less toxicity in the treatment of leukemia. Here, we report the evidence of delay leukemia relapse causing by a Cx43 pathway. The results suggest that modulation of Cx43 expression could be combined with current therapies to improve clinical outcomes on leukemia patients.

Gap junction channels are formed by two hemichannels (connexons), which are composed of six transmembrane proteins, con-nexins. There is at least 21 different human connexins have been reported [3] with a spectrum of homologs that manifest with various tissue or cell-specificity. Among these connexins, connexin 43 (Cx43) is considered as a major component of gap junctions in hematopoietic tissue [4]. Reduction or elimination of Cx43 containing gap junctions is frequently associated with tumor development [5–8], notably loss of gap junctional intercellular communication (GJIC) is an important step in carcinogenesis among various cancers [9]. Cx43 expression levels have been negatively correlated with tumor cell proliferation [10] and Cx43 over-expression in solid tumor suppresses abnormal cell proliferation [11, 12]. It also has been reported that lower Cx43 expression and GJIC function deterioration in bone marrow cells associated with leukemia development [9, 13]. It has been speculated that Cx43 expression in bone marrow stromal cells (BMSC) improves the GJIC between BMSC and leukemia cells in BM to limit leukemia cell proliferation. However, the mechanism of Cx43 regulated leukemia proliferation remains elusive. Here, we present evidence to show that Cx43 over-expression in BMSCs improves GJIC and induces apoptosis on leukemia cells through caspase 3 and 7.

In this study, we generated human Umbilical Cord Stem Cells (hUCSC) that over expressed Cx43 (Cx43- hUCSC). When Cx43-hUSCS co-cultured with L615 cell, a mouse T lymphoblastic leukemia cell line, GJIC is re-established on L615 with increasing apoptosis in L615 cells caused by Cx43-activating caspase 3 and caspase 7. In a minimal residual disease (MRD) mouse model, we further demonstrated that the relapse of leukemia was delayed by Cx43-hUCSCs transplantation. Previous studies have reported that Cx43 over-expression associates with apoptosis in breast cancer [14, 15] and improves drug sensitivity in glioblastoma [16]. Our data agreed with these observations and provide direct evidence that Cx43 acts through caspase 3 and 7 for inducing apoptosis in leukemia cells. The knowledge gained from this study could be used to facilitate the development of Cx43 modulation strategies in combination with current cancer therapies, such as small molecules including quinolines to target GJIC during carcinogenesis [10, 17, 18].

## RESULTS

### BMSC with over-expressed Cx43

**Figure 1:**
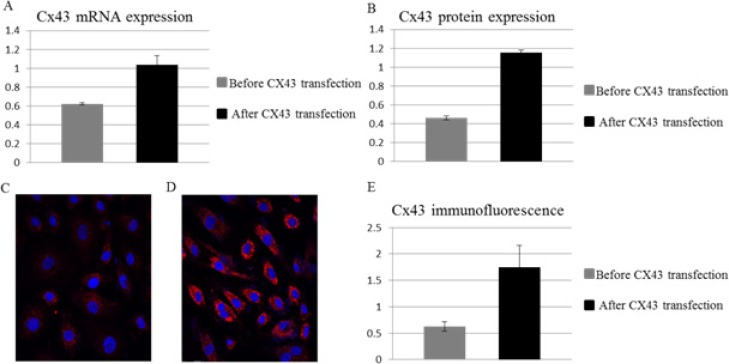
Over-expression of Cx43 on hUCSCs Mononuclear cells from human cord blood were obtained by ficoll density gradient and cultured for 2 days alone or with Cx43 vector for Cx43 over-expression (**A**) Quantification of Cx43 RNA expression normalized to β-Actin. (**B**) Quantification of Cx43 protein by western blot with antibodies against Cx43 and normalized to β-Actin. Immunofluorescence microscopy of transfected cells labeled with antibodies against Cx34 (red) before transfection (**C**) and 30 days after transfection (**D**). Nuclei were labeled with 4,6 diamidino-2-phenylindole (DAPI; blue). (**E**) Quantification of Cx43 labeling fluorescent intensity normalized to DAPI.

We have previously reported a Cx43 over-expression system on BMSCs with adenoviral vector [19]. This adenoviral vector was used to transfect hUCSC, a primary BMSC source, for stable expression of Cx43. The Cx43 expression are confirmed by semi-quantitative PCR for mRNA (Figure 1A) and by Western blot (Figure 1B) for protein expression at various time post-transfection. Immunofluorescence assay further confirm that Cx43 expression are significantly up-regulated after the introduction of Cx43 expression vector (Figure 1C–1E). While the Cx43 mRNA level is double post-transfection (Figure 1A), the protein expression of Cx43 is triple (Figure 1B) and stable for up to 30 days as reported previously [19]. Semi-quantitative immunostaining further confirmed that majority of cells are transfected (Figure 1D) and expression of Cx43 are triple comparing to expression before transfection (Figure 1E). This result indicates that Cx43-hUCSC has 3 fold higher Cx43 protein expression levels than that of un-manipulated hUCSCs.

### Cx43-hUCSC improves GJIC on L615 cells

**Figure 2:**
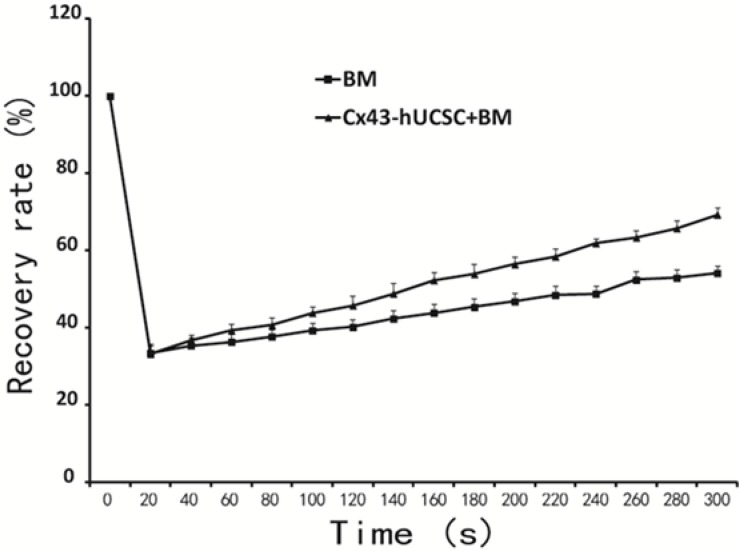
Cx43-hUCSC improves GJIC among cells L615 cells were cultured to 80% confluence on top of a mixture of L615 BM+Cx43-hUCSCs or on top of L615 BM only in a chamber slide. The dye CFDA, is loaded into the chamber (10 μmol/L, 15 min) before FRAP assay. Clusters of 5 to 6 cells (L615 cells) were selected under the microscope with the 20 × objective lens. Cells then were photo bleached to 10% of the original fluorescence intensity. Cell fluorescence intensity recovery was quantified after photo bleaching and plotted against time.

With BMSC over-expressing Cx43 (Cx43-hUCSC), we next evaluated the effect of increasing Cx43 on cell gap junction intercellular communication (GJIC). L615 cells were cultured alone or on top of Cx43-hUCSCs for three hours in chamber slides. The gap-FRAP (fluorescence recovery after photo bleaching) technique with 5(6)-carboxyfluorescein diacetate (CFDA) is used to measure the GJIC function as described previously [20, 21]. The gap-FRAP is noninvasive and faster than other approaches like dye transfer or scrape loading [22]. Our data show that 38.13% of L615 cells co-cultured with BM + Cx43-hUCSCs could recover at 1min after photo bleaching and 69.33% (± 1.25) cells recovered within 5 minutes. In contrast, only 34.7% of L615 cells cultured with BM alone had recovered fluorescence after 1 min with a maximal recovery percentage of 51.67% (± 1.7%) at 5 minutes. The recovery rate is significantly different at 5 minutes (300s) post photo bleaching (*p* < 0.01) (Figure 2). The result indicates that co-culture with Cx43-hUCSC improves GJIC function on L615 cells.

### Induction of apoptosis and alteration of cell cycle by Cx43-hUCSC

**Figure 3:**
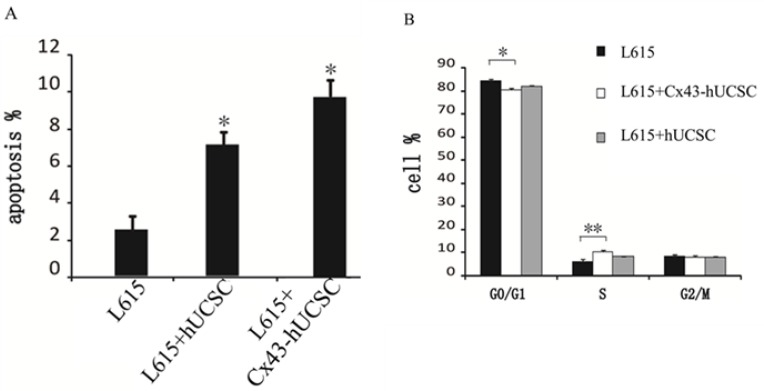
Cx43-hUCSC induces apoptosis and alters cell cycle profile L615 cells were cultured to 80% confluence and either by itself or co-cultured with hUCSC or with Cx43-hUCSC cells for three hours. (**A**) Apoptosis is quantified by fluorescent detection of FITC-labeled anti annexin V antibodies (*N* = 5) with FACS. (**B**) Annexin V negative L615 cells were sorted by flow cytometry and the cell cycle distribution (G0/G1, S and G2 + M phase) was quantified by Cell Cycle Assay Kit (Abcam, USA) and analyzed by Cell Fit 2.0 and 2.1 software. *indicates *p* < 0.05, **indicates *p* < 0.01.

We further evaluate the effect of Cx43 on L615 leukemia cells by co-culturing L615 with Cx43-hUCSC. L615 cell growth was measured 3 hours after co-culturing with Cx43-hUCSC or hUCSCs. Growth of L615 was inhibited by co-culturing (both Cx43-hUCSC or hUCSC) and agrees with previous reports [11, 19]. Both apoptosis and cell cycle arrest could lead to growth inhibition. We first investigate apoptosis rate by measuring annexin V on L615 cells with fluorescence-activated cell sorting (FACS). The percentages of cells undergo apoptosis (annexin positive cells) increased from 2.50 ± 0.85% to 7.33 ± 0.74% (*p* < 0.05) after co-culturing with hUCSCs. Co-culturing with Cx43-hUCSCs further increase the apoptosis rate to 9.70 ± 0.83% (*p* < 0.05) (Figure 3A). These data indicate that both co-culturing with hUCSC and increasing Cx43 expression on hUCSCs contribute to the increasing apoptosis in L615 cells. The Cx43 expression on BMSCs (Cx43-hUCSC) has synergy effect with BMSC co-culturing on L615 apoptosis.

We then performed cell cycle analysis on cells that did not undergo apoptosis (Annexin negative) with FACS (Figure 3B). Annexin negative cells are isolated and subjected to cell cycle analysis with Cell Cycle Assay Kit (Abcam, USA). Our results show that the majority of L615 cells (84%) remained in G0/G1 with a small percentage (6%) in S phase when they are cultured alone. When L615 cells are co-cultured with hUCSC or Cx43-hUCSC, the percentages of cells in G0/G1 phase decrease to 82% and 80% respectively. At the same time, the percentages of cells in S phase increase to 8% and 10% respectively. There is no significant difference among cells at G2/M phase under all three conditions (L615 alone, co-culture with hUCSC, and co-culture with Cx43-hUCSC). There is a statistically significant difference of cells in G0/G1 phase between L615 alone and L615 in co-culture conditions (with Cx43-hUCSC or with hUCSC) (*P* < 0.05).

For cells in S phase, there is a significant difference between L615 along and L615 co-cultured with Cx43-hUCSC (*P* < 0.01), but with less difference between L615 alone and L615 co-cultured with hUCSC (*P* < 0.05). These data indicate that while co-culturing with BMSC promotes cells entering the S phase from Go/G1 phase, Cx43 expression on BMSC further enhances this effect. This result agrees with the observation that Cx43 expression enhanced chemotherapy on glioblastoma [16] because cells at S phase is the target of many chemotherapy drugs.

### Cx43-hUCSC activates caspase pathways in L615 cells

**Figure 4:**
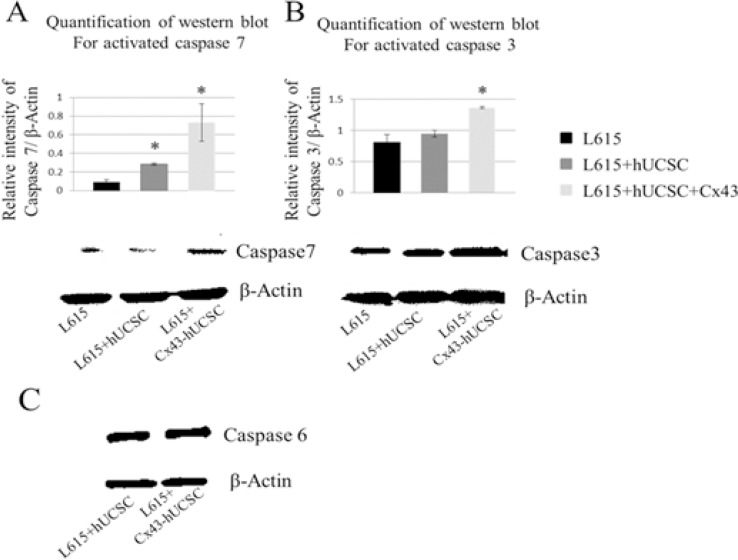
Increasing levels of activated caspase in L615 cells when co-cultured with BMSCs L615 cells were cultured alone, or co-cultured with either Cx43-hUCSC or hUCSC for three hours. Then L615 cells were harvested and the levels of activated caspase 3, 6 and 7 were quantified by Western blotting. (**A**) Levels of active caspase 7 were significantly increased in the conditions where cells were co-cultured with hUCSC or with Cx43-hUCSC compared to that of L615 cells grow alone. Activated caspase 7 was increased 3 fold in the hUCSC co-culture compared to that of L615 alone. When L615 cells are co-cultured with Cx43-hUCSC, active caspase 7 is increased up to 7 fold above that of L615 alone. Representative blots for Caspase 7 and β-Actin are shown. (**B**) Levels of active caspase 3 were significantly increased in L615 cells co-cultured with Cx43-hUCSC, but not in co-cultured with hUCSC. *indicates *p* < 0.05 (*N* = 3) (**C**) There is no difference in the levels of active caspase 6 among different culture conditions.

Although the observation of Cx43 inducing apoptosis on cancer cells has been reported [16, 19], the molecular mechanism of Cx43-induced apoptosis remains elusive. Therefore, we investigate one of the major molecular pathways of apoptosis, the caspase pathway. After co-cultured with hUCSCs or Cx43-hUCSCs for 3 hrs, L615 cells were harvested from the adherent hUCSC or Cx43-hUCSC layer. The levels of activated/cleaved effector caspases 3, 6 and 7 were measured by western blot for 3 conditions: L615 (cultured alone), L615 co-cultured on hUCSC or on Cx43-hUCSC (Figure 4). A substantially higher level of active caspase 3 and 7 are detected in the co-cultured conditions (with hUCSC and Cx43-hUCSC). Active caspase 7 levels were three fold higher in hUCSC co-cultures than that of L615 alone (Figure 4A). Active caspase 7 levels were increased up to seven fold than that of L615 alone when L615 co-cultured with Cx43-hUCSC. Levels of active caspase 3 also increased in both co-culture conditions (Figure 4B). However, they were significantly higher in the case where cells were co-cultured with Cx43-hUCSC comparing to that of L615 alone (1.8 fold increase). There were no significant differences in the levels of activated caspase 6 among all 3 conditions (L615 alone, hUCSC co-culture, and Cx43-hUCSC co-culture) (Figure 4C). Both hUCSC and Cx43-hUCSC expressed Cx43, but Cx43-hUCSC expressed 3 fold higher of Cx43 than that of hUCSC. Collectively, these data suggest that Cx43 has different effects on different caspases and the effect is not linear with Cx43 expression levels on hUCSC. These data also suggest that Cx43 induced apoptosis on L615 may be due to the activation of caspase 3 and 7, but not caspase 6.

### Cx43-hUCSC transplantation

**Figure 5:**
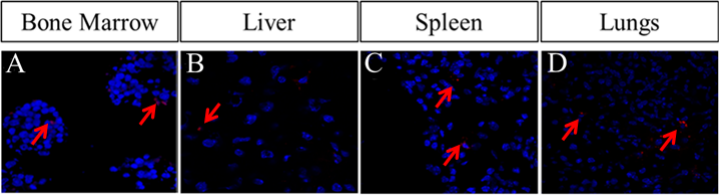
Homing of Cx43-hUCSCs to bone marrow, liver, spleen, and lung of L615 mouse The Cx43-hUCSCs were labeled with CM-Dil (red) and 1 × 10^6^ Cx43-hUCSC mixed with 1 × 10^6^ bone marrow cells from L615 mice were injected into L615 mouse via tail veins. Images are fluorescence tracing of CM-Dil labeled Cx43-hUCSC in Bone marrow (**A**) liver (**B**) spleen (**C**) and lungs (**D**) post transplantation. Cell nuclei were labeled with 4, 6 diamidino-2-phenylindole (DAPI; blue).

After assessing effect of Cx43-hUCSC *in vitro* with co-culture system, we further investigate the effect of Cx43-hUCSC in an animal model of leukemia. We first tested the engraftment of Cx43-hUCSC and their migration to bone marrow, liver, spleen and lungs. We used CM-DIl (Life Technologies) to label Cx43-hUCSC before transplantation into mouse via tail vein. CM-DIl is a dye for labeling viable cells and its intensity will be diluted with cell division (decreasing by two folds for each division). Fluorescent imaging of labeled Cx43- hUCSCs shows CM-DII positive cells in L615 mouse bone marrow on day 14 after transplantation (Figure 5A). From day 1 to day 14, while the number of fluorescent cells increased, the fluorescence intensity of individual cells was decreased. Labeled Cx43-hUCSCs also shows engraftment of cells in L615 mouse liver (Figure 5B) spleen (Figure 5C,), and lungs (Figure 5D). These data suggest that the transplanted cells are viable in mouse up to 14 days and actively dividing.

### Cx43 expression increases survival rate of leukemia mouse

**Figure 6:**
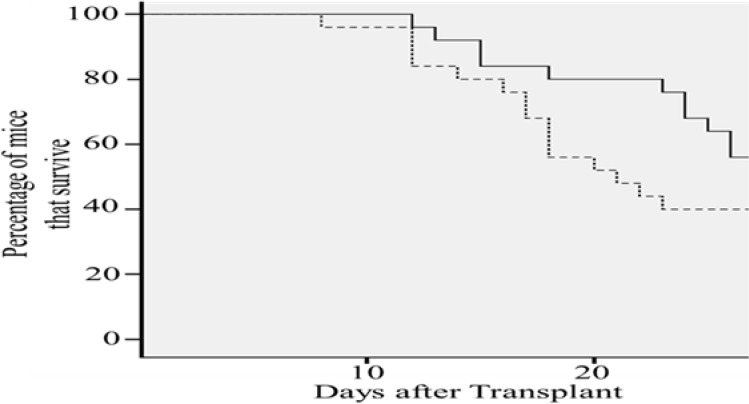
Cx43-hUCSC transplantation increases survival time of mice with minimal residual disease (MRD) L615 mouse MRD model were generated by injection of L615 leukemia cells into L615 mouse followed with chemotherapy (CTX, 200 mg/kg). Two weeks of CTX treatment reduce the L615 cell to an undetectable level. Transplantations with Cx43-hUCSC (1 × 10^6^) mixed with BM (1 × 10^6^) or BM alone (2 × 10^6^) were performed via tail vein two weeks post CTX treatment. Mortality rate of injected mice was monitored for 30 days after the injections and was calculated to be 15% lower in mice transplanted with Cx43-hUCSC than that with BM alone. The average survival time of mice injected with Cx43-hUCSC (27 days after transplant) is significantly longer than that of the BM group (23 days).

The transplanted Cx43-hUCSCs not only viable after transplantation, but also delay the relapse of leukemia and improve the survival rate in a MRD mouse model. A mouse MRD model was generated by transplanting L615 leukemia cells into L615 mouse and following with chemotherapy cyclophosphamide (CTX, 200 mg/kg) to reduce the L615 leukemia cells to a minimal residual level. This mouse model mimics the typical leukemia relapse in patients with MRD. After transplantation of Cx43-hUCS +BM or BM alone, survival of L615 mice was monitored for 28 days (Figure 6). We found that the average mortality rate of mice with Cx43-hUCSCs + BM transplantation was 15% lower than that of mice undergone BM transplantation only (*p* < 0.01). For the mouse transplanted with Cx43-hUCSC+BM, the mean survival time is 26.9 days post-transplant. For the mice transplanted with BM only, the mean survival time is 23.7 days. These data indicate that Cx43-hUCSC can delay the relapse of leukemia and improve the survival rate in mice with MRD.

## DISCUSSION

Traditionally leukemia therapy targets leukemia cells with chemo- or radio-ablation. However, the side effect and toxicity of such approaches significantly affects the quality of life in many patients and leads to patient deaths due to the therapy rather than the leukemia. Recently, more and more studies suggest that only targeting the leukemia cells is not sufficient. Approaches targeting the leukemia microenvironment could be in combination with standard therapy to improve clinical outcomes. One of such approaches is targeting gap junction intercellular communication (GJIC) between leukemia cells and bone marrow stromal cells (BMSCs) for improving clinical outcomes of leukemia. Loss of GJIC is a common feature during carcinogenesis [10, 17] in various cancer. It has been reported in many studies of solid tumors that restoring GJIC by Cx43 expression can inhibit tumor cell growth [10, 17, 20, 23]. However, the mechanism of Cx43 acting as a tumor suppressing gene in leukemia has not been fully studied and remains elusive. Here, we report evidence to show that Cx43 expressed by BMSC induces apoptosis on leukemia cells via the caspase 3 and caspase 7 pathways.

With an established *in vitro* co-culture system [19, 24] and a MRD mouse model, we investigate the effects of Cx43 expressed by BMSCs in preventing leukemia relapse. In this study, CX43 was over-expressed on hUCSC (a stable BMSC resource) and co-cultured with L615 leukemia cells. As expected, hUCSC co-culture inhibits L615 growth, and Cx43-hUCSC (hUCSC over expressed Cx43) inhibits L615 cell proliferation. Fluorescent recovery rate after photo-bleaching (FRAP) indicated that Cx43-hUCSC improves GJIC between L615 and BMSC. Co-culturing L615 on top of Cx43-hUCSC increases apoptosis of L615 more than that of hUCSC co-culture. Further analysis of caspase pathways in L615 indicate that caspase 3 and caspase 7, but not caspase 6 were activated by Cx43 expression from the hUCSCs.

The caspase family of cysteine proteases, have long been associated with stress induced, program cell death (PCD) and inflammatory responses. The efficacy of caspases is decidedly dependent on the specific stimulus that triggered it, as well as genetic and epigenetic factors [25] and a growing amount of biochemical evidence now demonstrates that caspase 3, 6 and 7 define a diversity of non-overlapping substrates that mediate specific aspects of PCD. Our results show that activated caspase 3 and 7, but not caspase 6 are significantly increased in L615 cells when co-culture with Cx43-hUCSC. Besides its role in apoptosis activation, caspase 7 has been also observed in inflammation process that seem to require activation of lysosomes and direct interaction with activator caspase 1 [26]. Even though in our experiments we see a robust increase of active caspase 3, we find that the activation of caspase 7 is much better defined. This result suggests that the apoptotic response could be graded and or exhibiting a higher signaling threshold before caspase 3 is recruited. The details of how caspase 3 and 7 intersect and interact with Cx43 GJIC mediated apoptosis still unclear. Our study is the first step to investigate this process.

In this study, we reported that Cx43-hUCSCs induce apoptosis on leukemia cells through activation of caspase 3 and 7 but not caspase 6. Cx43 expression by BMSC can promote leukemia cells (L615 cells) to enter S phase and these S phase cells are the primary target of most chemotherapy drugs. We further show that Cx43-hUCSC can delay relapse and improve survival rate in a MRD mouse model. These results shine lights on the mechanism of Cx43 induction of tumor apoptosis. Small molecules such as quinolones have been reported for manipulating Cx43 expression. These small molecules could be developed into complemental drugs for leukemia therapy. Using small molecules (e.g. quinolones) to increase Cx43 expression on BMSC has the potential to supplement current chemotherapy in improving clinical outcomes of leukemia.

## MATERIALS AND METHODS

### L615 cell lines and mice

L615 cell lines were purchased from the institute of Academy of Medical Sciences (Tianjin, China) and routinely maintained in PRMI1640 with 10% (v/v) fetal bovine serum (Hyclone, USA). All animal protocols were approved by the University Institutional Animal Care and Use Committee (IACUC). All methods were carried out in accordance with the approved guidelines and protocols. Female L615 mice (aged 4–6 weeks, 22–29 g body weight) were purchased from the Chinese Academy of Medical Sciences & Peking Union Medical College Laboratory Animal Center. Animals were housed in specific pathogen–free (SFP) rooms.

### Culture of hUCSCs and bone marrow cells from L615 mice

Cord bloods were taken from umbilical cords of full-term normal-delivery babies delivered by healthy mothers. Written informed consent was obtained from mothers in all cases with approval from the ethics committee. Heparin was used as the anti-coagulant. The hUCSC were harvested as described previously [24]. In brief, mononuclear cells from human cord blood were obtained by Ficoll density gradient fractionation columns (density = 1.077 g/l Pharmacia Biotech, Uppsala, Sweden). Cells were re-suspended in DMEM medium (Gibco, USA) supplemented with 12.5% (v/v) fetal bovine serum (Hyclone, USA), 12.5% (v/v) horse serum (Gibco, USA), 10−6 M hydrocortisone, 10 ng/ml SCF (Sigma, USA), and 1 ng/ml bFGF (Sigma, USA) [34]. BMSCs of L615 mice were separated from femur and tibia of L615 mice, and were plated in T25 flasks for continuous passage in F12 (Hyclone, USA) medium supplemented with 10% FBS (Gibco, USA). Medium was changed every 3 days, and cells were passaged into fresh flasks at a ratio of 1: 4 upon reaching confluence using trypsin (Hyclone, USA).

### Detection of Cx43 expression in hUCSCs after transfection

Adenovirus vector containing Cx43 gene sequence was transfected into hUCSCs as previously described [19]. PCR was used for determination of Cx43 mRNA expression levels. In brief, total RNA was isolated with Trizol reagent (Invitrogen, USA) according to the manufacturer's instructions. The cDNA synthesis and amplification via PCR were performed using the PrimeScript^®^ 1st Strand cDNA Synthesis Kit (Takara, Japan) on a Mycycler Thermal Cycler (Bio-Rad, USA). Gene expression (from 50 ng cDNA) was measured by qPCR using the Ex Taq kit (Takara, Japan) and custom primers for Cx43: F: 5′-AACCTGGTTGTGAAAATGTC-3′; R: 5′-GCAAGTGTAAACAG CACTCA-3′ and β-actin (NM 001101): F: 5′- CCTGTGGCATCCACGAAACT-3′; R: 5′-GAGCAATGATCCTGATCTTC-3′.

### Immunofluorescence

Cx43-hUCSCs were cultured on glass cover slips in six well plates and grown to 80% confluence. Cells were washed with PBS and fixed with 40 mg/L paraformaldehyde (Sigma, USA) for 25 min, then blocked with 10% goat serum albumin (Sigma, USA) for 20 min before staining. A drop of DAPI staining solution (Lifetechnologies, USA, 1 mg/ml) was added to stain the cells for 5 min. The slips of cells were washed with PBS, and a drop of 1:100 diluted mouse anti-human Cx43 monoclonal antibody (Abcam, USA) was added and incubated for 24 h at 4°C. Samples were warmed to room temperature for 1 h, washed with PBS, 1 drop of 1:100 diluted FITC-labeled goat anti-mouse IgG (Abcam, USA) was added and incubated for 1 h at room temperature. Mouse monoclonal IgG1 (Abcam, USA) was used as isotype control as instructed by manufactures. After washing with PBS, the slips were sealed using 60% buffering glycerol (Sigma, USA) and imaged by a laser confocal microscope (Leica, Germany) with a fast scanning mode.

### Cells proliferation assay and cell cycle analysis

The Cx43-hUCSCs were seeded at 1 × 10^5^ cells/well in a 6-well plate. CFDA labeled L615 cells (Vybrant^®^, ThermoFisher, USA) were then co-cultured on top of the Cx43-hUCSC layer 24 hr after seeding and forming of Cx43-hUCSC monolayer. Cell proliferation assay was performed using the Cell Counting Kit-8 (Dojindo Japan). At different time points, CFDA + L615 cells were harvested by selective trypsinization (after PBS washing off non-binding cells, 0.25% trypsin was added to disassociate L615 cells from Cx43-hUCSC monolayer) and further isolated from hUCSC by FACS. Single-cell suspension (100ul of a 5 × 10^4^ cells/ml) was added into a 96-well plate with triplicate wells for each group. The cells were cultured at 37°C for 3 h after adding 10 ul reagent from Cell Counting Kit-8. For cell cycle analysis, the harvested L615 cells were washed twice with ice-cold PBS, fixed with 75% cold ethanol (4°C) over night. The cell cycle phase was analyzed by FACS flow cytometry. The cell cycle distribution (G0/G1, S and G2 + M) was quantified using the Cell Fit 2.0 and 2.1 software (Becton Dickinson).

### Establishment of a minimal residual disease (MRD) model

L615 cells (1 × 10^5^ cells, labeled with GFP viral vector) were injected into L615 mouse via tail veins for development of leukemia. After detection of GFP + L615 cells in peripheral blood, cyclophosphamide (CTX) (200 mg/kg) was injected into the peritoneal cavity three days later to suppress the L615 leukemia cells, and produce a model for minimal residual disease after treatment (~1% of GFP L615 in bone morrow). All animal experiments were approved by the ethics committee.

### Cx43-hUCSCs migration in mouse

For labeling live Cx43-hUCSCs, the cells were incubated at 37°C in CM-DiI solution (2ug/ml) for 5 minutes, and then for an additional 15 minutes at 4°C. After labeling, cells were washed with PBS twice and resuspended in fresh medium before transplantation into L615 mouse via tail veins. BM group was infused with 2 × 10^6^ bone marrow cells from L615 mice and Cx43-hUCSCs + BM group was infused with 1 × 10^6^ Cx43-hUCSCs and 1 × 10^6^ bone marrow cells from L615 mice. Following the injection, transplanted L615 mice were housed in SFP animal rooms. Mice were sacrificed by neck dislocation. Frozen sections (8 μm) of fresh frozen Liver, spleen, and lung tissue were prepared and fixed in frozen acetone for five minutes. The sections were then stained with DAPI and examined with fluorescence microscope. The Hemapun 948 cold vacuum embedding technique was used to prepare Bone marrow sections [2] : Bone marrow pathological tissues was fixed in pre-cooled 4°C Bouin fixative solution for 1 h, washed with 0.1 ml/L sodium dimethylarsinate buffer in a 4°C refrigerator for 30 min, and dehydrated at 4°C in a gradient of alcohol concentrations for 10 min in each concentration. Living tissue blocks were then placed on the bottom of a mold with 2 ml of solution A in a type 1354 vacuum dryer for 2–4 h at 4°C. Two to three drops of alcohol were then added into the mold with living tissue blocks at 4°C to make the complete embedding solution and the polymerization was completed under vacuum conditions.

### Fluorescence recovery after photo bleaching (FRAP) assay

GJIC was quantitatively assessed in living cells by FRAP assay as previously described [22]. In brief, the L615 cells were monitored for transfer of fluorescent dye from neighboring cells and examined for recovery of fluorescence by scanning at intervals of 50 s for a total period of 300 s. The maximum intensity of recovered fluorescence (It) at the 300th s was collected as the functional index of GJIC. At least five such clusters were selected from each dish. The analyzed fluorescence recovery index is expressed as: *R* = (It−I0)/(I−I0) × 100%, where I0 is the intensity of the photobleached fluorescence, and I is the intensity of pre-bleached fluorescence.

### Statistical analysis

Data was presented as mean values with standard deviation. Statistical significance was analyzed with the Student's *t* test. *P* values less than 0.05 were considered statistically significant.
